# A Scientometric Approach to Review the Role of the Medial Preoptic Area (MPOA) in Parental Behavior

**DOI:** 10.3390/brainsci11030393

**Published:** 2021-03-20

**Authors:** Alessandro Carollo, Jan Paolo Macapinlac Balagtas, Michelle Jin-Yee Neoh, Gianluca Esposito

**Affiliations:** 1 Department of Psychology and Cognitive Science, University of Trento, 38068 Rovereto, Italy; alessandro.carollo@studenti.unitn.it; 2 Psychology Program, School of Social Sciences, Nanyang Technological University, Singapore 639798, Singapore; JANP0001@e.ntu.edu.sg (J.P.M.B.); MICHELLE008@e.ntu.edu.sg (M.J.-Y.N.); 3 Lee Kong Chian School of Medicine, Nanyang Technological University, Singapore 636921, Singapore

**Keywords:** medial preoptic area, MPOA, parental behavior, scientometry, systematic review, citespace, document co-citation analysis, keyword analysis

## Abstract

Research investigating the neural substrates underpinning parental behaviour has recently gained momentum. Particularly, the hypothalamic medial preoptic area (MPOA) has been identified as a crucial region for parenting. The current study conducted a scientometric analysis of publications from 1 January 1972 to 19 January 2021 using CiteSpace software to determine trends in the scientific literature exploring the relationship between MPOA and parental behaviour. In total, 677 scientific papers were analysed, producing a network of 1509 nodes and 5498 links. Four major clusters were identified: “C-Fos Expression”, “Lactating Rat”, “Medial Preoptic Area Interaction” and “Parental Behavior”. Their content suggests an initial trend in which the properties of the MPOA in response to parental behavior were studied, followed by a growing attention towards the presence of a brain network, including the reward circuits, regulating such behavior. Furthermore, while attention was initially directed uniquely to maternal behavior, it has recently been extended to the understanding of paternal behaviors as well. Finally, although the majority of the studies were conducted on rodents, recent publications broaden the implications of previous documents to human parental behavior, giving insight into the mechanisms underlying postpartum depression. Potential directions in future works were also discussed.

## 1. Introduction

Across many species, social encounters and interactions are ubiquitous and the regulation of social behaviours is essential for health and survival. With the advent of neurobiological methods, researchers are able to investigate the neural basis underlying social behaviour, gaining insight into processes of the brain that govern social behaviour. Among the wide range of social behaviours, this paper will focus on the study of parental behaviour and its neurobiological basis.

As young in mammalian species are usually altricial at birth, parental care is often a critical aspect for the survival and development of offspring. Parental behaviours form a complex category of activities influenced by a range of internal and external factors [1], where laboratory rodents are popular animal models used to study these factors. In rodents, general responses can be categorized into nurturance, indifference/avoidance and infanticide. Specifically, parental behaviours include active behaviours such as nest construction, pup retrieval and licking of pups and quiescent behaviours such as quiescent positioning over pups (see Lonstein and Fleming [2]). Sex differences are observed in parenting behaviours where male and female rodents differ in spontaneity of parental behaviours. While both virgin and postpartum female mice are spontaneously maternal and have an innate motivation to care for pups [2,3], virgin males often engage in infanticide where they attack and kill newborn pups as an adaptive reproductive strategy to increase their mating opportunities [4,5,6,7]. On the other hand, male mice only become parental in the weeks following mating [6]. Similarly, female rats are (i) less likely to be infanticidal [7,8,9], (ii) more spontaneously responsive to pups or likely to become parentally sensitized [10,11,12] and (iii) more consistent in displaying particular parental behaviours [13,14].

In terms of the neurobiology underlying parental behaviours in rodents, the medial preoptic area (MPOA) of the hypothalamus—an area involved in thermoregulation and sexual behaviour—is one of the key areas which has been implicated and is often considered a central node in the control of parenting. Empirical studies found (i) lesions in the MPOA disrupted parental behaviour [15], (ii) high expression of receptors of modulators of parenting such as estrogen, oxytocin, progesterone and prolactin [16], (iii) facilitation of parental behaviour when the MPOA is directly stimulated with estrogen [17,18]. Galanin-expressing neurons has also been found to govern parental behaviour in mice. Loss of galanin neurons in the MPOA was associated with a reduction in parental behaviour in male and female mice while optogenetic activation of galanin neurons reduced pup-directed aggression and induced active pup grooming in male mice [19].

## 2. The Present Study

Considerable progress in identifying brain areas and neural mechanisms underlying parenting has been made in the last few decades (see [20,21] for reviews). Given the size of the body of this work, the present study used a scientometrics approach to investigate influential and impactful publications in this field through document co-citation analysis (DCA; e.g., [22]), where a co-citation is defined as the citation of two sources together in the same paper. The DCA will show clusters of co-citing publications and papers and temporal and structural metrics of these clusters will be discussed. Specifically, we aim to investigate: (i) common topics of the clusters of publications co-citing each other and (ii) impactful publications. In order to accomplish both aims, a DCA was computed and, especially for investigating the common topics and main trends within the literature, it was supported by an analysis of keywords and indexing terms. The results of the scientometric approach will provide greater insight into research trends and publications in the field of the neurobiology of parenting behaviour.

## 3. Materials and Methods

**Figure 1:**
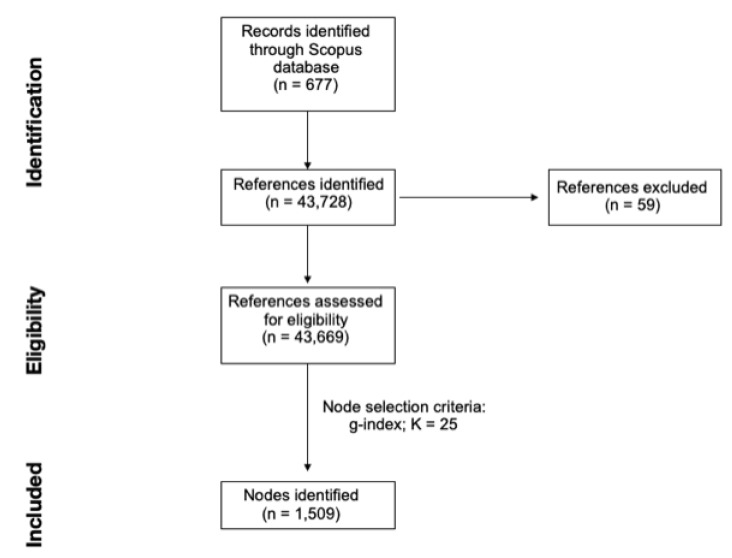
Study flow diagram.

For the present study, the sample of publications was downloaded from the database on Scopus, as done in previous scientometric publications [23,24]. A total of 677 scientific works, published between 01 January 1972 to 19 January 2021, was available on Scopus when using the following research string for the search: “( TITLE-ABS-KEY ( “medial preoptic” ) OR TITLE-ABS-KEY ( “MPOA” ) AND TITLE-ABS-KEY ( parent* ) OR TITLE-ABS-KEY ( matern* ) OR TITLE-ABS-KEY ( patern* ) OR TITLE-ABS-KEY ( mother* ) OR TITLE-ABS-KEY ( attach* ) OR TITLE-ABS-KEY ( nurtur* ) OR TITLE-ABS-KEY ( offspring ) OR TITLE-ABS-KEY ( pup* ) OR TITLE-ABS-KEY ( attack* ) OR TITLE-ABS-KEY ( infanticid* ) OR TITLE-ABS-KEY ( “young” )) AND (LIMIT-TO( LANGUAGE, “English”))”. After downloading the dataset of publications, scientometric analysis was conducted via CiteSpace software (version 5.7.R3). Data was imported in CiteSpace and 43,669 of the 43,728 (99.87%) total references cited in the collected papers were considered valid. A loss of a small percentage of references (~1.0–5.0%), when importing data in the software, is typically due to data irregularities that cannot be processed by CiteSpace. The amount of invalid references can be considered as negligible (Figure 1) [25].

To examine the connections between scientific works investigating the relationship between medial preoptic area and parenting, a DCA was first conducted. DCA is a type of analysis based on the frequency in which two documents have been co-cited (cited together) by subsequent works [26]. On CiteSpace, this type of analysis is conducted not only by considering the documents that are downloaded from scientific databases, but also by analyzing the references that they have cited in their text. Therefore, the DCA generates a network which consists in both citing (the ones downloaded from scientific platforms) and cited documents. In the analysis, the g-index selection criteria was adopted with scale factor k set at 25. The selection criteria and the value for its scale factor were chosen after several trials in which we aimed to optimize the structural metrics of the network. In particular, we attempted the analysis with g-index with k set at 25 and 15, TOP N with N at 50, 15 and 10 and TOP N% with N at 10. These three types of selection criteria rely on different principles to select the nodes that compose the network. The first one, g-index, which is based on the h-index, is the “largest number that equals the average number of citations of the most highly cited g publications” [27]. Conversely, TOP N and TOP N% criteria select the N or N% most cited nodes within a given time slice to form the network. The DCA was subsequently supported by a keyword analysis and the same optimization of node selection criteria was conducted. In this case, the best selection criteria turned out to be TOP N with N fixed at 10.

Structural metrics were used to examine the overall configuration of the network and the details of each node. Structural metrics include modularity Q, silhouette score and betweenness centrality. Modularity Q is an index that ranges from 0 to 1 and indicates the extent to which a network is divisible into single modules or clusters [28]. The homogeneity of these modules is measured using the silhouette score, with values ranging from −1 to 1. The higher the value of silhouette score, the higher the consistency of nodes among the module [29,30]. Betweenness centrality applies to single nodes to describe the degree in which a single node functions as a bridge to connect other nodes which would otherwise be separate. Centrality values range from 0 to 1, where high scores close to 1 indicates likely groundbreaking ideas [24]. For the analysis of single nodes, alongside the already mentioned structural metrics, temporal metrics were examined as well. This group of metrics mainly refers to citation burstness and sigma. Citation burstness is an index that indicates an abrupt change in the frequency in which a node has been cited within a period of time [31]. On CiteSpace, values of citation burstness are computed following Kleinberg’s algorithm [32]. Theoretically, values of citation burstness can range from 0 to infinite. Sigma is a metric obtained by considering betweenness centrality and citation burstness at the same time. Sigma values are computed following the Equation (centrality+1)${}^{burstness}$ [29], and they indicate the novelty and the influence of a node among the network of interest.

## 4. Results

### 4.1. Document Co-Citation Analysis

The network we obtained for the DCA was composed of 1509 nodes and 5498 links. This means that, on average, each node in the network was connected with 3.64 other references. Furthermore, the network showed a modularity Q index of 0.3841 and a weighted mean silhouette of 0.9257. Thus, the nodes form a network which is modestly divisible into separate modules, each of which is highly homogeneous.

**Figure 2:**
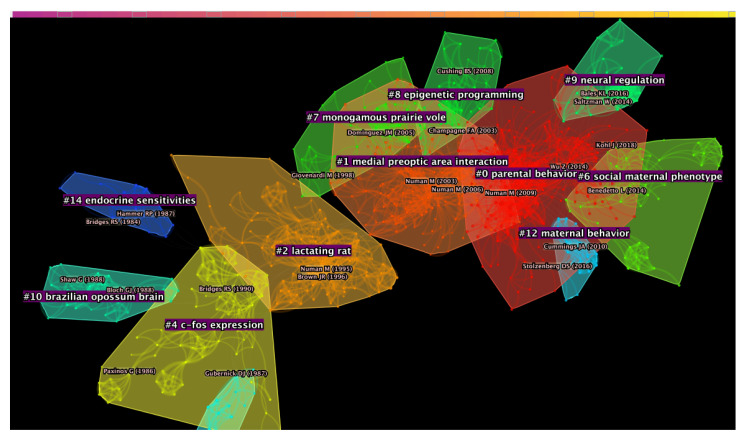
Network of publications generated through the Document Co-Citation Analysis (DCA). The 17 major clusters are highlighted and divided by colour.

The major clusters identified in the DCA were highly internally homogeneous (see Figure 2 and Table 1). The largest cluster, cluster #0, that was identified consisted of 190 nodes, had a silhouette score of 0.879 and the references composing it were, on average, published in 2010. Cluster #1 was a group of 129 nodes with a high silhouette score of 0.885 and a publication year that, on average, was 2002. The third largest cluster, that is cluster #2, was a group of 115 nodes with a high silhouette score of 0.902 and were on average, published in 1994. The next cluster, cluster #4, consisted of 69 nodes, had silhouette of 0.915 and mean publication year of 1990. Considering the average year of publication of the documents forming a cluster, cluster #6 was the most recent one (mean year of publication = 2014; size = 50; silhouette = 0.968) together with cluster #9 (mean year of publication = 2014; size = 36; silhouette = 0.99).

In the network, 55 nodes showed a citation burst in their history (see Table 2 for the 20 nodes with the highest value of citation burstness). In particular, among the 10 nodes with the highest magnitude of citation burstness, 6 belonged to cluster #0, 3 to cluster #1 and 1 to cluster #2. Specifically, among the three documents with the strongest magnitude of citation burst, 1 belonged to cluster #1 and 2 to cluster #0. In order of burst strength, these three references were authored by Numan and Insel [16], Wu et al. [19] and Numan and Stolzenberg [33]. The document authored by Numan and Insel [16] showed a citation burst of 18.61, which started in 2005, two years after its publication. Wu et al. [19] were the authors of the publication with the second highest citation burst in the network, with a value of 16.05 and a duration of 5 years. Finally, the third document with the strongest citation burst, specifically 14.55, was authored by Numan and Stolzenberg [33]. Among the 20 references reported in Table 2, M. Numan was the first author for 7 of the references. Among these, the node by Numan et al. [34] had the longest citation burst in the network, with a duration of 8 years (from 2005 to 2013). As for the sigma metric, the document with the highest value was again the one published by Numan and Insel [16], with a value of 3.02. Other references with high sigma values were authored by Numan and Stolzenberg [33] and Champagne et al. [35], with sigma values of 2.29 and 1.91, respectively.

### 4.2. Keywords Analysis

The Keywords Analysis produced a network of 55 nodes and 161 links, meaning that each identified keyword was connected with 2.93 other ones. Furthermore, the resulting network showed a modularity Q index of 0.2488 and a weighted mean silhouette of 0.7898. In the network, 22 nodes showed a citation burst (see Table 3). In particular, by looking at the strength of their citation burst, *support*, *metabolism* and *physiology* had the highest magnitudes of 49.07, 42.12 and 33.13, respectively. *Support* is a keyword used especially as an indexing term in the Medical Subject Headings (MeSH) database, and it refers to the funding of the research. Therefore, such keyword is not relevant for the understanding of the trends in the research on the role of the MPOA in parenting. As for the duration of the citation burst, two keywords were particularly relevant: *rat* (strength of burstness = 7.15; burst duration = 22 years) and *hypothalamus* (strength of burstness = 24.34; burst duration = 21 years). *Hypothalamus* was also the keyword with the earliest beginning of citation burst, which started in 1972. The other references with the earliest beginning of burstness were *theoretical study* (beginning of burstness = 1974) and *rat* (beginning of burstness = 1977). conversely, the keywords with a more recent citation burst were *metabolism* (beginning of burstness = 2015), *physiology* (beginning of burstness = 2015), *maternal behavior* (beginning of burstness = 2016) and *male* (beginning of burstness = 2018).

## 5. Discussion

### 5.1. Document Co-Citation Analysis

The content of the major clusters, whose titles were given using the Log-Likelihood Ratio (LLR) option, identified through the DCA is discussed below. Specifically, clusters are presented following the chronological order in which their documents were, on average, published.

#### 5.1.1. Cluster #4: “C-Fos Expression”

In Table 4, the most active citing documents for cluster #4 are reported. c-Fos is an immediate early response gene encoding a transcription factor that is part of the AP-1 transcription factor complex, which is involved in the regulation of cell proliferation. c-Fos has also been found to be a marker of neuronal activity (see [51]). In particular, as the name of the cluster suggests, some references in the cluster focused on understanding the underlying mechanisms of parental behavior by examining Fos-like immunoreactivity (Fos-lir) in the brain. This approach allowed researchers to find that MPOA is a crucial area for the onset and maintenance of parental behavior. In fact, the MPOA has a greater number of cells showing Fos-lir in maternally active rats [52,53,54]. Within this cluster, the onset of maternal behavior in rats was also examined in relation to lactogen and the central administration of human placental lactogen. Specifically, the work by Bridges and Freemark [55] and Bridges et al. [56] showed that human placental lactogen infusion in the MPOA of steroid-primed nulliparous rats facilitates the onset of maternal behaviors towards a foster young, similar to what was reported for prolactin [57,58,59,60]. For this reason, some references in which authors explored the functional and structural properties of the response to prolactin were included in cluster #4 [61,62,63,64,65,66]. The role of estrogens has also been studied by part of the references of this cluster in regards to maternal behavior in rodents [67,68,69]. For instance, the study by Rosenblatt et al. [70] showed that the MPOA is crucial for maternal behavior even in male rats and that such behavior benefits from a prolonged estradiol and progesterone treatment. This interest found in the citing documents justifies the presence of papers studying the localization and distribution of estrogen receptors in various animals’ brains, such as rodents [71] and quails [72] within the cluster. The study of estrogens and the MPOA, occasionally with a focus on aromatase action, permitted researchers to explore the sexual dimorphism reported for this brain area as well [73,74,75,76,77,78,79].

#### 5.1.2. Cluster #2: “Lactating Rat”

In Table 5, the most active citing documents for cluster #2 are reported. As the name of the cluster suggests, part of the references in this group studied the relationship between MPOA and parental behavior by focusing on lactating rats. For this reason, some works within the cluster are cited because they explore the brain response to prolactin, the levels of which increase during lactation [82,83,84,85,86,87,88,89]. Other hormones related to this phase have been studied as well [90,91,92]. For instance, the increased binding of oxytocin in MPOA at parturition seems to be important for the molecule to stimulate the postpartum activation of maternal behavior [93]. This onset of maternal behavior after parturition depends on lateral habenula neurons [94,95,96]. As observed in the previous cluster, the MPOA is a brain area that shows an increase in the immediate early gene c-fos or other Fos proteins in maternally active rats [47,53,54,97,98,99,100,101,102]. Such activation of the MPOA appears to have a causal role for the ability to nurture young animals [45,70]. Thus, references composing cluster #2 aimed to better understand some properties of such responses [103,104,105,106,107]. For instance, Lin et al. [108] showed that FosB and Fos in the MPOA (and other areas) are involved in the neural activation during parturition and lactation, not in pregnancy, in rats. Furthermore, a part of that neural activation seems to be independent from olfactory and other sensory inputs, indicating the presence of efferent neurons crucial for the performance of maternal behavior [38,109]. Furthermore, Lonstein and De Vries [110] reported that many of the neurons showing c-fos activity after maternal behavior are GABAergic. For this reason, the authors concluded that some neurons in the MPOA, especially in its dorsal part, and other brain areas (i.e., the ventral bed nucleus of the stria terminalis and the caudal ventrolateral periaqueductal gray) must have either a local or long-range inhibitory effect as a component of maternal behavior. Knowledge of the presence of a circuit of inhibiting maternal behavior within the hypothalamus and in connected brain regions started to emerge from studies of the beginning of 2000s [111,112]. In the same years, Komisaruk et al. [113] reported that in parturient and hysterectomized rats, there is an increase in excitatory interactions in the MPOA. By examining Fos expression during maternal behavior, Stack et al. [43] observed that the MPOA likely modulates the activity of two brain regions: the shell of the nucleus accumbens, and the intermediate part of the Lateral Septum. Another work by Lonstein et al. [114] documented that a number of Fos-immunoreactive (Fos-ir) neurons also express the alpha subtype of the estrogen receptor (ERα), suggesting that postpartum maternal behavior could be influenced by ERα activity [17]. In fact, [115] noted that MPOA’s susceptibility towards the effects of estrogen increases right after pregnancy termination. To highlight the connection between estrogens and Fos-ir neurons in MPOA, c-fos expression inducted in rodents’ brain by estradiol administration has been reported in the literature [116,117]. In fact, evidence of the central role that such hormones have on maternal behavior comes from studies on the administration of estrogen-progesterone treatment (to simulate a pregnancy-like pattern of hormonal environment) to nonpregnant and ovariectomized rats. These animals were still able to manifest maternal behaviours with treatments simulating the hormonal pattern of pregnancy [118].

#### 5.1.3. Cluster #1: “Medial Preoptic Area Interaction”

In Table 6, the most active citing documents for cluster #1 are reported. As suggested by the name of the cluster, and anticipated by the previous one, the interest of researchers in those years was oriented towards expanding the focus of attention towards a circuit, and not only a single area, controlling parental behavior. For these reasons, researchers started to look at the interactions between the MPOA and other brain regions in order to better understand the regulation of parental behavior [120]. To do so, Numan et al. [34] hypothesized that the way in which the MPOA facilitates maternal behavior in rats involves circuits of inhibition [121]. In fact, the MPOA forms connections with the nucleus accumbens, which exerts inhibitory GABAergic control over the ventral pallidum, a central region involved in eliciting maternal responses in response to pup stimuli. For the authors, the MPOA facilitates maternal behavior by inhibiting the nucleus accumbens and, therefore, indirectly activating the ventral pallidum. In support of the role of the nucleus accumbens in maternal behavior, the study by Olazabal and Young [122] showed that oxytocin receptors in this brain region, whose expression increases in the MPOA and other areas after parturition [123,124,125,126], is related to the expression of spontaneous maternal behavior in prairie voles. In the same way, dopamine D1 receptors antagonists disrupt retrieval and licking of pups in rats when injected in the nucleus accumbens [46,127,128], and also in the MPOA [129]. The region of the nucleus accumbens critical for pup-retrieval behavior seems to be the shell [130], which seems to be involved in the consolidation of maternal memory [131,132]. Even with some subtle differences, dopamine receptor antagonists modify parental behavior even in prairie voles [133]. Dopamine in the nucleus accumbens was also linked to rats’ maternal behavior [134], specifically, pup licking/grooming [35]. Based on this evidence, some authors suggested that the neural system controlling maternal behavior in rats could overlap with the dopamine circuit of rewards in the brain [135,136,137,138]. The neural model designed to explain the mechanisms with which the MPOA controls maternal behavior included two paths of actions [37]. In the first one, the activated MPOA would inhibit a central aversion system responsible for defensive and avoiding behaviors towards pups. In the second, the MPOA would act by exciting the mesolimbic dopamine system in order to promote voluntary maternal responses [139,140,141]. Therefore, some references within the cluster were cited because they explored the properties of the dopamine mesolimbic circuit [142,143,144,145,146,147,148]. The neural model of maternal behavior was refined in the review written by Numan and Stolzenberg [33]. Here, the authors discussed the interaction between the dopamine system and the MPOA [149]. In particular, they reported findings suggesting that the MPOA activates the shell region of the nucleus accumbens through mesolimbic dopaminergic inputs in order to control aspects of maternal appetitive behavior [43]. To facilitate the effect of the MPOA on the nucleus accumbens, dopamine from the incerto-hypothalamic system interacts with steroid and peptide hormones to finally act on the MPOA [150,151]. For this reason, part of the references in the cluster were cited because they studied the effects of steroid or peptide hormones on parental behavior [115,119,152,153,154,155,156,157,158,159]. As a matter of fact, some of these molecules seem to be crucial for maternal aggression aimed at protecting offspring [160,161,162,163,164,165]. If dopamine levels in MPOA seem to increase during lactation [166], the neural origin of such molecular inputs was debated. For instance, Miller and Lonstein [167] did not find a significant number of dopaminergic terminals arriving at the MPOA from the zona incerta of the brain, but found them in other brain regions, such as the ventrocaudal posterior hypothalamus, the medial supramammillary nucleus and part of the ventral tegmental area. In fact, the causal role of the ventral tegmental area, a crucial area in the mesolimbic circuit whose activity is regulated by GABAergic and glutamatergic connections from the bed nucleus of the stria terminalis [168,169,170], in maternal behavior is documented by Numan et al. [171]. In this regard, a temporary inactivation of the ventral tegmental area in postpartum female rats interferes with the preference for pup-paired context in a conditioned place preference paradigm and reduced pup licking and pup retrieval behaviors [172]. In the same way, the inhibition of the medial prefrontal cortex negatively affected the pup retrieval behavior in maternal rats [173]. The motivational perspective on the female’s response to her offspring started to grow following the trend of research in the 2010s. It became clear that in that period, immediately after parturition, several brain structures (including the MPOA) contribute towards inducing a pup-specific bias to the motivational circuitry [15,41,174,175].

#### 5.1.4. Cluster #0: “Parental Behavior”

In Table 7, the most active citing documents for cluster #0 are reported.

In particular, Rutherford et al. [178] followed the approach of research suggesting the involvement of the reward system on parental behavior [48,134,179,180]. By using a place preference method, Mattson and Morrell et al. [181] found that the MPOA was the only area showing a larger activation when dams preferred pup-associated versus cocaine cues, a preference that has been replicated in the literature [182,183]. In this rewarding process, oxytocin is a molecule that, for its role in social cognition and social rewards [184], plays a role in the stimulation of dopamine in the mesolimbic system, making child stimuli more rewarding [40,185]. During the 2010s, it became evident that maternal experience also has a role in regulating behaviors targeted at caring for offspring [186]. For instance, the dopaminergic response to pup-exposure in the shell of the nucleus accumbens depends on the female’s experience with pups, with higher experience associated with higher levels of dopamine [187]. In fact, the mesolimbic pathways sustain the changes due to maternal experience, with both dopamine receptor subtypes in the nucleus accumbens allowing the consolidation of this experience-dependent memory [188]. Olazabal et al. [189], by proposing new models to explain maternal behavior in different species and contexts, highlighted the flexible role of the MPOA in such neural circuits, an area that seems to facilitate maternal behavior during the early postpartum period and inhibit it in the later postpartum [190]. This transient role within the motivational system that the MPOA plays in the regulation of parental behavior is also detected in the available literature on the topic [41]. A final aim of the work by olazabal et al. [189] was to extend the knowledge obtained from other species to human mothering. This intent, as in other works in the literature [191], was pursued also by Lonstein et al. [192], who compared the evidence on the biopsychological influences that regulate maternal behaviors obtained from studies on animal models (mainly rats and sheep) to extend the understanding of human maternal behavior. The authors of this review reported many similarities and differences in factors influencing mothering among species. The differences would be linked to species-specific features, such as the role of hormones, of each sensory system, the flexibility in behavior, whether there is a language or not, and the role of cortical functions. These evidence led many researchers to explore the mechanisms underlying postpartum neuropsychiatric disorders, which are reported by many women. In particular, the review written by Mchenry et al. [193] studied the changes in reproductive steroids in order to activate maternal behavior and their association with postpartum neuropsychiatric disorders in many women, especially affective disorders [194,195]. The authors suggest that many brain regions, including the MPOA and the ventral bed nucleus of the stria terminalis, could mediate these effects for their influences on motivation and anxiety during the postpartum period [193,196]. This influence of the MPOA and the bed nucleus of the stria terminalis appears to depend on maternal experience [177]. In fact, maternal memory, which in part depends on amygdaloid V1a receptors [197] and the nucleus accumbens shell [198], is known for influencing the female’s behaviors towards pups in rats [199]. Furthermore, following the trends of research investigating neural plasticity mainly in the MPOA and the hippocampus [200,201,202,203,204], Pawluski and Galea [205] and Pawluski et al. [206] showed that the properties of the hippocampus vary during pregnancy and mothering. An insight on postpartum mood disorders following alterations of the maternal neural systems was also given by other references in the cluster [42,207,208].

Another trend of research within the cluster looked at the fact that lactating dams are less fearful than non-maternal animals and they exhibit lower hypothalamic-pituitary-adrenal (HPA) activation in response to potential environmental threats [209]. The diminished responsiveness of the HPA axis, which leads to a general sense of calmness in mothers, are due to the modified activity within two systems: a circuit that inhibits the HPA axis (e.g., oxytocin and prolactin systems) and another one with excitatory effects on the HPA axis. The first one would see an increased activation during lactation, whereas the second one would see a reduction in its activity [210]. The review by Bosch [211] was focused on the role that the reduction of anxiety in lactation plays in maternal behavior. In fact, high innate anxiety in dams tends to lead to intense and protective maternal behavior alongside an increased aggression towards a virgin intruder. Such behavior is considered functional to protect the pup against infanticide. Oxytocin and vasopressin are involved in this process reported in the review [44,212,213,214]. As a matter of fact, the release of these molecules in areas such as the hypothalamus and the limbic system contributes to the regulation of maternal behavior, including maternal anxiety and aggression [150,165,215,216,217,218,219,220,221,222,223,224,225,226]. For this interest, some references within the cluster were cited because they studied the mechanisms of action of those molecules [49,227,228,229,230,231,232]. Specifically, the mother’s brain sees an increased release of oxytocin during breastfeeding. When functional magnetic resonance imaging is used on dams, the brain’s pattern of activation following administration of oxytocin overlaps with the pattern of activation during pup suckling. This pattern included brain regions known for their role in regulating olfactory discrimination, emotions and reward [233]. Moreover, pup suckling activates multisensory processes in the brain of lactating dams [234,235]. In the review by Dobolyi et al. [236], authors focused on the role of the input from pups that activate the MPOA and, therefore, maternal behavior. The authors discussed that, in rodents, neurons containing the tuberoinfundibular peptide of 39 residues in the posterior thalamus appear to be good candidates to convey the suckling information to the MPOA, supporting maternal responsiveness. The way in which these inputs influence the neurons in the MPOA seems to depend on modifications of the gene expression, which seem to support maternal behavior [237,238,239]. In particular, chromatin remodelling mediates these alterations in the long-term [240,241].

Within this cluster, Tsuneoka et al. [36] studied the subregions in the MPOA that are necessary for maternal behavior in mice. Their results showed that the central part of the MPOA is crucial for maternal behavior, for its lesions lead to infanticide. Moreover, this subregion of the MPOA shows a c-fos activation during maternal behavior mostly in regards to GABAergic and peptidergic (galanin, neurotensin, and/or tachykinin2 mRNAs) neurons, as in other studies [19]. Alongside this interest on deepening the understanding of the role of the MPOA in female parental behaviors, some authors within the cluster tried to examine whether such knowledge could be applied to males’ parental behavior [242,243]. As a matter of fact, males do not undergo the hormonal cascade at a female’s parturition, postpartum ovulation and lactation. Nevertheless, in new fathers, the MPOA, the bed nucleus of the stria terminalis and the caudal dorsal raphe nucleus are activated in response to pups [244,245]. As in females, paternal experience in rodents is associated with a diminished activation in fear/anxiety brain regions and an increased activity in the network responsible for affiliative responses, which included the MPOA [246]. Upon becoming parents, male rodents have to inhibit the mechanisms responsible for infanticide and activate direct parental behavior towards the offspring. Evidence showed that the downregulation of the activity of neurons along the vomeronasal system, mainly sensitive to non-volatile chemosensory stimuli, is responsible for the shift from aggression towards pups in sexually naive male mice to the display of paternal behavior in fathers [247]. Nevertheless, virgin males’ aggressive behavior guided by the activity of the vomeronasal system seems to rely on the morphological features of the pup and salivary chemosignals [248]. Similar to female rodents, the central part of the MPOA plays a crucial role in parental behavior in male rodents, alongside the bed nucleus of stria terminalis. In fact, Tsuneoka et al. [50] showed that the activity in these two brain regions was able to classify paternal and infanticidal motivation with high accuracy (95%). Furthermore, lesions of the central MPOA induced infanticide, whereas lesions of the rhomboid nucleus of the bed nucleus of stria terminalis showed the opposite pattern, inhibiting infanticidal behaviors in virgin males. Moreover, the expression of androgen receptor and estrogen receptor (ER) α and their interaction with testosterone is fundamental for males’ sexual, aggressive and parental behaviors. This expression depends on the neuronal subtypes and the subregions examined in the MPOA [249].

### 5.2. Keywords Analysis

The analysis of the keywords with a citation burst supports the trends of research that emerged from the analysis of publications composing the network’s clusters. In particular, besides the keywords indicating the brain region of interest for the specific studies (central nervous system, hypothalamus, medial preoptic area, preoptic area and brain), the other indexing terms mostly referred to the methodology used to investigate the relationship between the MPOA and parenting. As observed from the results, the vast majority of studies in the field was conducted on rats using methods to analyze protein expression within the brain. Furthermore, as emerged from the discussion on clusters, most of the studies focused on the period of time around pregnancy (pre- and postpartum). Furthermore, although the majority of the studies exploring the role of the MPOA in parenting were conducted on females, research interest has recently involved males and paternal behavior as well.

## 6. Conclusions

In this paper, we used a scientometric analysis to identify research trends in past publications investigating the association between the MPOA and parental behaviour. We found four major clusters that characterised significant time periods in this dynamic area of research in order of chronology: “C-Fos Expression", “Lactating Rat", “Medial Preoptic Area Interaction" and “Parental Behavior". Initial research interest focused on examining an established physiological response in the brain associated with the maternal response (first cluster) and then, mainly by the study of lactating rats (second cluster), new models to explain maternal behavior were conceptualised. Subsequently, equipped with insight gathered from these new models, researchers turned their focus towards investigating brain circuitry beyond just the MPOA in controlling parental behaviour (third cluster). Finally, the most recent predominance in research looks at disentangling the many aspects of parental behaviour, including paternal behavior and infanticide (fourth cluster). The keyword analysis also identified terms, especially those associated with brain regions and methodology, that extend beyond the clusters, revealing possible foundations for subsequent research contributing to this line of work.

This study has some limitations which we outline here. Firstly, The publication sampling method employed specifies a constraint of identified keywords to be present in the titles, which, though it has the obvious advantage of streamlining the selection process, excludes relevant publications which may not have been captured [252]. Consequently, underlying themes in the research not reflected in the publication titles, abstract or keywords may have been overlooked. Next, only a few keywords were used to extract source articles from a single database, Scopus. Extensions of this work may consider using alternative keywords and including other databases (e.g., Web of Science) to determine whether the pattern of results hold. This specific scientometric approach may also be unable to fully encapsulate the extent of the trends in this research, particularly in view of unpublished works [253].

Another limitation of the scientometric approach is that it strongly relies on citations among documents. This method gives insight into the trends of research, but it does not capture the specific nature of the relations among co-cited papers. Analysing citations among documents leads to a bias towards past publications. In fact, documents published in the past tend to have higher number of citations as compared to more recent ones. This difference in the number of citations between past and recent publications would not reflect a difference in importance and scientific impact, but a difference in the “years of life" of a publication. A final and more technical limitation is that the references within the file downloaded from Scopus were not always consistent in the way they are cited by other publications. Sometimes this leads CiteSpace to consider two formats of citation of the same reference as two different publications. For this reason, some documents appear more than once within a cluster or even within the network. As regards to the four main clusters that were discussed, only a small number (3.4%) of references within the four main clusters that were discussed in the present article were repeated. Being this rate negligible, we have preferred not deleting them from the original file to not lose useful information.

The role the MPOA plays in parental behaviour continues to be a dynamic area of research. Historically, there has been a large focus on parental behaviour in mothers, particularly in the pregnancy or lactating stages of motherhood, for the role of the hormones associated with those phases. As seen in the fourth cluster, there is a growing trend in research investigating infanticide and paternal behaviour. Future work may build on this relatively recent development and use the findings in this paper as a guide in the literature exploring the role of the MPOA on parental behaviour. This line of research in paternal behaviour could lead to the elucidation of the underlying basis of sex differences in nurturing behaviors among parents, particularly sexual dimorphisms in the brain. Nonetheless, continual work in examining the neural correlates of maternal behaviour has relevant practical implications in understanding the driving factors predisposing many women to experience neuropsychiatric conditions in the postpartum period, namely depression [254].

## Figures and Tables

**Table 1:** Metrics of the 10 largest clusters identified by computing the Document Co-Citation Analysis (DCA).

Cluster ID	Size	Silhouette	Mean Year
0	190	0.879	2010
1	129	0.885	2002
2	115	0.902	1994
4	69	0.915	1990
6	50	0.968	2014
7	41	0.966	2001
8	39	0.983	2004
9	36	0.99	2014
10	29	0.989	1987
11	27	0.983	1987

**Table 2:** Description of the identifying characteristics of the 20 publications with high citation burstness metrics generated in the DCA.

Reference	Strenght of Burstness	Year	Beginning of Burstness	End of Burstness	Burst Duration	Sigma	Centrality
Numan and Insel [16]	18.61	2003	2005	2011	6	3.02	0.06
Wu et al. [19]	16.05	2014	2015	2020	5	1.84	0.04
Numan and Stolzenberg [33]	14.55	2009	2010	2017	7	2.29	0.06
Tsuneoka et al. [36]	13.67	2013	2014	2020	6	1.49	0.03
Numan [37]	10.95	2006	2007	2013	6	1.71	0.05
Numan and Numan [38]	10.24	1995	1997	2002	5	1.16	0.01
Dulac et al. [39]	9.58	2014	2015	2020	5	1.19	0.02
Shahrokh et al. [40]	8.94	2010	2011	2017	6	1.48	0.04
Pereira and Morrell [41]	8.90	2011	2013	2020	7	1.29	0.03
Numan et al. [34]	8.26	2005	2005	2013	8	1.58	0.06
Bridges [42]	7.39	2015	2016	2020	4	1.00	0.00
Stack et al. [43]	7.20	2002	2005	2010	5	1.28	0.04
Bosch and Neumann [44]	7.19	2012	2013	2020	7	1.11	0.01
Champagne et al. [35]	7.09	2004	2005	2012	7	1.91	0.10
Brown et al. [45]	6.99	1996	1997	2002	5	1.41	0.05
Keer and Stern [46]	6.59	1999	2004	2007	3	1.18	0.03
Numan and Numan [47]	6.30	1994	1998	2002	4	1.21	0.03
Numan [48]	5.92	2007	2008	2015	7	1.05	0.01
Meddle et al. [49]	5.83	2007	2010	2014	4	1.01	0.00
Tsuneoks et al. [50]	5.82	2015	2017	2020	3	1.01	0.00

**Table 3:** Main characteristics of the twenty-two keywords with the generated citation burstness metrics in the Keywords Analysis.

Reference	Strength of Burstness	Beginning of Burstness	End of Burstness	Burst Duration
*support*	49.07	1979	1995	16
*metabolism*	42.12	2015	2020	5
*physiology*	33.13	2015	2020	5
*central nervous system*	28.94	1978	1987	9
*hypothalamus*	24.34	1972	1993	21
*medial preoptic area*	14.02	2010	2020	10
*maternal behavior*	11.07	2016	2018	2
*pregnancy*	10.05	1978	1985	7
*male*	8.59	2018	2020	2
*protein expression*	7.90	2002	2010	8
*preoptic area*	7.82	2004	2008	4
*rat*	7.15	1977	1999	22
*estradiol*	6.55	1987	1989	2
*radioisotope*	6.54	1984	1990	6
*animal tissue*	5.57	2009	2011	2
*endocrine system*	5.37	1982	1984	2
*ovariectomy*	5.24	1987	1989	2
*theoretical study*	4.82	1974	1977	3
*histology*	4.67	1980	1982	2
*aging*	4.55	1978	1989	12
*brain*	4.24	1987	1988	1
*animal cell*	3.92	1987	1989	2

**Table 4:** All the 9 citing documents in cluster #4 identified using the DCA.

Cluster	Citing Document	GCS	Coverage
4	Nagano and Shinoda [80]	20	9
4	Bridges et al. [56]	78	9
4	Fleming and Walsh [53]	79	9
4	Fleming and Korsmit [54]	108	8
4	Dellovade et al. [79]	32	7
4	Rizvi et al. [81]	135	7
4	Rosenblatt et al. [70]	75	6
4	Bridges and Freemark [55]	34	6
4	Ehret and Buckenmaier [69]	36	5

**Table 5:** Major 10 citing documents in cluster #2 identified using the DCA.

Cluster	Citing Document	GCS	Coverage
2	Kalinichev et al. [102]	54	21
2	Stack et al. [43]	103	15
2	Lonstein et al. [114]	72	14
2	Komisaruk et al. [113]	31	13
2	Sheehan and Numan [115]	48	13
2	Stack and Numan [107]	56	13
2	Grattan [119]	114	12
2	Lin et al. [108]	29	12
2	Li et al. [101]	88	12
2	Lonstein and De Vries [110]	64	11

**Table 6:** Major 10 citing documents in cluster #1 identified using the DCA.

Cluster	Citing Document	GCS	Coverage
1	Gammie [120]	69	25
1	Curtis et al. [176]	57	19
1	Numan [37]	159	17
1	Numan and Stolzenberg [33]	224	17
1	Numan et al. [128]	119	15
1	Numan and Woodside [174]	89	15
1	Pereira and Morrell [41]	84	14
1	Perrin et al. [177]	37	14
1	Numan et al. [34]	91	14
1	Olazabal and Young [122]	176	12

**Table 7:** Major 10 citing documents in cluster #0 identified using the DCA.

Cluster	Citing Document	GCS	Coverage
0	Lonstein et al. [192]	62	31
0	Numan and Young [250]	131	23
0	Mchenry et al. [193]	20	20
0	Olazabal et al. [189]	55	18
0	Dobolyi et al. [236]	40	18
0	Yoshihara et al. [226]	15	18
0	Rutherford et al. [178]	81	17
0	Bosch [211]	100	16
0	Bridges [42]	146	16
0	Numan [251]	0	16

## Data Availability

Data will be available upon request.

## Abbreviations

The following abbreviations are used in this manuscript:

DCA	Document co-citation analysis
Fos-ir	Fos-immunoreactive
Fos-lir	Fos-like immunoreactivity
HPA	hypothalamic–pituitary–adrenal
MPOA	Medial preoptic area

## References

[1] Rosenblatt J., Lehrman D. (1963). Maternal Behavior in Mammals.

[2] Lonstein J.S., Fleming A.S. (2001). Parental behaviors in rats and mice. Curr. Protoc. Neurosci..

[3] Numan M. (2014). Neurobiology of Social Behavior: Toward an Understanding of the Prosocial and Antisocial Brain.

[4] Trivers R.L. (1972). Parental investment and sexual selection. Sexual Selection & the Descent of Man, Aldine de Gruyter, New York.

[5] Elwood R. (1977). Changes in the responses of male and female gerbils (Meriones unguiculatus) towards test pups during the pregnancy of the female. Anim. Behav..

[6] Vom Saal F.S. (1985). Time-contingent change in infanticide and parental behavior induced by ejaculation in male mice. Physiol. Behav..

[7] Mennella J.A., Moltz H. (1988). Infanticide in the male rat: The role of the vomeronasal organ. Physiol. Behav..

[8] Jakubowski M., Terkel J. (1985). Incidence of pup killing and parental behavior in virgin female and male rats (*Rattus norvegicus*): Differences between Wistar and Sprague-Dawley stocks. J. Comp. Psychol..

[9] Brown R.E. (1986). Social and hormonal factors influencing infanticide and its suppression in adult male Long-Evans rats (*Rattus norvegicus*). J. Comp. Psychol..

[10] Quadagno D.M., Rockwell J. (1972). The effect of gonadal hormones in infancy on maternal behavior in the adult rat. Horm. Behav..

[11] Fleischer S., Kordower J.H., Kaplan B., Dicker R., Smerling R., Ilgner J. (1981). Olfactory bulbectomy and gender differences in maternal behaviors of rats. Physiol. Behav..

[12] Samuels M.H., Bridges R.S. (1983). Plasma prolactin concentrations in parental male and female rats: Effects of exposure to rat young. Endocrinology.

[13] Mayer A.D., Freeman N., Rosenblatt J.S. (1979). Ontogeny of maternal behavior in the laboratory rat: Factors underlying changes in responsiveness from 30 to 90 days. Dev. Psychobiol. J. Int. Soc. Dev. Psychobiol..

[14] Gray P., Chesley S. (1984). Development of maternal behavior in nulliparous rats (*Rattus norvegicus*): Effects of sex and early maternal experience. J. Comp. Psychol..

[15] Lee A., Clancy S., Fleming A.S. (1999). Mother rats bar-press for pups: Effects of lesions of the mpoa and limbic sites on maternal behavior and operant responding for pup-reinforcement. Behav. Brain Res..

[16] Numan M., Insel T.R. (2003). The Neurobiology of Parental Behavior.

[17] Rosenblatt J.S., Ceus K. (1998). Estrogen implants in the medial preoptic area stimulate maternal behavior in male rats. Horm. Behav..

[18] Rosenblatt J.S., Olufowobi A., Siegel H.I. (1998). Effects of pregnancy hormones on maternal responsiveness, responsiveness to estrogen stimulation of maternal behavior, and the lordosis response to estrogen stimulation. Horm. Behav..

[19] Wu Z., Autry A.E., Bergan J.F., Watabe-Uchida M., Dulac C.G. (2014). Galanin neurons in the medial preoptic area govern parental behaviour. Nature.

[20] Kohl J., Autry A.E., Dulac C. (2017). The neurobiology of parenting: A neural circuit perspective. Bioessays.

[21] Kohl J., Dulac C. (2018). Neural control of parental behaviors. Curr. Opin. Neurobiol..

[22] Chen C., Song I.Y., Yuan X., Zhang J. (2008). The thematic and citation landscape of data and knowledge engineering (1985–2007). Data Knowl. Eng..

[23] Maia S.C., de Benedicto G.C., do Prado J.W., Robb D.A., de Almeida Bispo O.N., de Brito M.J. (2019). Mapping the literature on credit unions: A bibliometric investigation grounded in Scopus and Web of Science. Scientometrics.

[24] Aryadoust V., Zakaria A., Lim M.H., Chen C. (2020). An extensive knowledge mapping review of measurement and validity in language assessment and SLA research. Front. Psychol..

[25] Chen C. (2016). CiteSpace: A Practical Guide for Mapping Scientific Literature.

[26] Gaggero G., Bonassi A., Dellantonio S., Pastore L., Aryadoust V., Esposito G. (2020). A scientometric review of alexithymia: Mapping thematic and disciplinary shifts in half a century of research. Front. Psychiatry.

[27] Egghe L. (2006). Theory and practise of the g-index. Scientometrics.

[28] Aryadoust V., Tan H.A.H., Ng L.Y. (2019). A Scientometric review of Rasch measurement: The rise and progress of a specialty. Front. Psychol..

[29] Chen C., Ibekwe-SanJuan F., Hou J. (2010). The structure and dynamics of cocitation clusters: A multiple-perspective cocitation analysis. J. Am. Soc. Inf. Sci. Technol..

[30] Chen C., Song M. (2019). Visualizing a field of research: A methodology of systematic scientometric reviews. PLoS ONE.

[31] Chen C. (2017). Science mapping: A systematic review of the literature. J. Data Inf. Sci..

[32] Kleinberg J. (2003). Bursty and hierarchical structure in streams. Data Min. Knowl. Discov..

[33] Numan M., Stolzenberg D.S. (2009). Medial preoptic area interactions with dopamine neural systems in the control of the onset and maintenance of maternal behavior in rats. Front. Neuroendocrinol..

[34] Numan M., Numan M.J., Schwarz J.M., Neuner C.M., Flood T.F., Smith C.D. (2005). Medial preoptic area interactions with the nucleus accumbens–ventral pallidum circuit and maternal behavior in rats. Behav. Brain Res..

[35] Champagne F.A., Chretien P., Stevenson C.W., Zhang T.Y., Gratton A., Meaney M.J. (2004). Variations in nucleus accumbens dopamine associated with individual differences in maternal behavior in the rat. J. Neurosci..

[36] Tsuneoka Y., Maruyama T., Yoshida S., Nishimori K., Kato T., Numan M., Kuroda K.O. (2013). Functional, anatomical, and neurochemical differentiation of medial preoptic area subregions in relation to maternal behavior in the mouse. J. Comp. Neurol..

[37] Numan M. (2006). Hypothalamic neural circuits regulating maternal responsiveness toward infants. Behav. Cogn. Neurosci. Rev..

[38] Numan M., Numan M.J. (1995). Importance of pup-related sensory inputs and maternal performance for the expression of Fos-like immunoreactivity in the preoptic area and ventral bed nucleus of the stria terminalis of postpartum rats. Behav. Neurosci..

[39] Dulac C., O’Connell L.A., Wu Z. (2014). Neural control of maternal and paternal behaviors. Science.

[40] Shahrokh D.K., Zhang T.Y., Diorio J., Gratton A., Meaney M.J. (2010). Oxytocin-dopamine interactions mediate variations in maternal behavior in the rat. Endocrinology.

[41] Pereira M., Morrell J.I. (2011). Functional mapping of the neural circuitry of rat maternal motivation: Effects of site-specific transient neural inactivation. J. Neuroendocrinol..

[42] Bridges R.S. (2015). Neuroendocrine regulation of maternal behavior. Front. Neuroendocrinol..

[43] Stack E.C., Balakrishnan R., Numan M.J., Numan M. (2002). A functional neuroanatomical investigation of the role of the medial preoptic area in neural circuits regulating maternal behavior. Behav. Brain Res..

[44] Bosch O.J., Neumann I.D. (2012). Both oxytocin and vasopressin are mediators of maternal care and aggression in rodents: From central release to sites of action. Horm. Behav..

[45] Brown J.R., Ye H., Bronson R.T., Dikkes P., Greenberg M.E. (1996). A defect in nurturing in mice lacking the immediate early gene fosB. Cell.

[46] Keer S., Stern J. (1999). Dopamine receptor blockade in the nucleus accumbens inhibits maternal retrieval and licking, but enhances nursing behavior in lactating rats. Physiol. Behav..

[47] Numan M., Numan M.J. (1994). Expression of Fos-like immunoreactivity in the preoptic area of maternally behaving virgin and postpartum rats. Behav. Neurosci..

[48] Numan M. (2007). Motivational systems and the neural circuitry of maternal behavior in the rat. Dev. Psychobiol. J. Int. Soc. Dev. Psychobiol..

[49] Meddle S.L., Bishop V.R., Gkoumassi E., van Leeuwen F.W., Douglas A.J. (2007). Dynamic changes in oxytocin receptor expression and activation at parturition in the rat brain. Endocrinology.

[50] Tsuneoka Y., Tokita K., Yoshihara C., Amano T., Esposito G., Huang A.J., Yu L.M., Odaka Y., Shinozuka K., McHugh T.J. (2015). Distinct preoptic-BST nuclei dissociate paternal and infanticidal behavior in mice. EMBO J..

[51] Baum M., Everitt B. (1992). Increased expression of c-fos in the medial preoptic area after mating in male rats: Role of afferent inputs from the medial amygdala and midbrain central tegmental field. Neuroscience.

[52] Numan M., Numan M. (1993). Fos production in preoptic neurons correlated with different aspects of maternal behaviour in rats. Soc. Neurosci. Abs.

[53] Fleming A.S., Walsh C. (1994). Neuropsychology of maternal behavior in the rat: C-fos expression during mother-litter interactions. Psychoneuroendocrinology.

[54] Fleming A.S., Korsmit M. (1996). Plasticity in the maternal circuit: Effects of maternal experience on Fos-Lir in hypothalamic, limbic, and cortical structures in the postpartum rat. Behav. Neurosci..

[55] Bridges R.S., Freemark M.S. (1995). Human placental lactogen infusions into the medial preoptic area stimulate maternal behavior in steroid-primed, nulliparous female rats. Horm. Behav..

[56] Bridges R.S., Robertson M.C., Shiu R.P., Friesen H.G., Stuer A.M., Mann P.E. (1996). Endocrine communication between conceptus and mother: Placental lactogen stimulation of maternal behavior. Neuroendocrinology.

[57] Kinsley C.H., Bridges R.S. (1988). Prolactin modulation of the maternal-like behavior displayed by juvenile rats. Horm. Behav..

[58] Stern J.M. (1989). Maternal behavior: Sensory, hormonal, and neural determinants. Psychoendocrinology.

[59] Bridges R.S., Numan M., Ronsheim P.M., Mann P.E., Lupini C.E. (1990). Central prolactin infusions stimulate maternal behavior in steroid-treated, nulliparous female rats. Proc. Natl. Acad. Sci. USA.

[60] Bridges R.S., Ronsheim P.M. (1990). Prolactin (PRL) regulation of maternal behavior in rats: Bromocriptine treatment delays and PRL promotes the rapid onset of behavior. Endocrinology.

[61] Walsh R.J., Mangurian L.P., Posner B.I. (1990). The distribution of lactogen receptors in the mammalian hypothalamus: An autoradiographic analysis of the rabbit and rat. Brain Res..

[62] Emanuele N., Jurgens J., Halloran M., Tentler J., Lawrence A., Kelley M. (1992). The rat prolactin gene is expressed in brain tissue: Detection of normal and alternatively spliced prolactin messenger RNA. Mol. Endocrinol..

[63] Hnasko R.M., Buntin J.D. (1993). Functional mapping of neural sites mediating prolactin-induced hyperphagia in doves. Brain Res..

[64] Buntin J.D., Ruzycki E., Witebsky J. (1993). Prolactin receptors in dove brain: Autoradiographic analysis of binding characteristics in discrete brain regions and accessibility to blood-borne prolactin. Neuroendocrinology.

[65] Sugiyama T., Minoura H., Kawabe N., Tanaka M., Nakashima K. (1994). Preferential expression of long form prolactin receptor mRNA in the rat brain during the oestrous cycle, pregnancy and lactation: Hormones involved in its gene expression. J. Endocrinol..

[66] Dutt A., Kaplitt M.G., Kow L.M., Pfaff D.W. (1994). Prolactin, central nervous system and behavior: A critical review. Neuroendocrinology.

[67] Giordano A.L., Siegel H.I., Rosenblatt J.S. (1989). Nuclear estrogen receptor binding in the preoptic area and hypothalamus of pregnancy-terminated rats: Correlation with the onset of maternal behavior. Neuroendocrinology.

[68] Giordano A.L., Siegel H.I., Rosenblatt J.S. (1991). Nuclear estrogen receptor binding in microdissected brain regions of female rats during pregnancy: Implications for maternal and sexual behavior. Physiol. Behav..

[69] Ehret G., Buckenmaier J. (1994). Estrogen-receptor occurrence in the female mouse brain: Effects of maternal experience, ovariectomy, estrogen and anosmia. J. Physiol.-Paris.

[70] Rosenblatt J.S., Hazelwood S., Poole J. (1996). Maternal behavior in male rats: Effects of medial preoptic area lesions and presence of maternal aggression. Horm. Behav..

[71] Cintra A., Fuxe K., Harfstrand A., Agnati L., Miller L. (1986). On the cellular localization and distribution of estrogen receptors in the rat tel-and diencephalon using monoclonal antibodies to human estrogen receptor. Neurochem. Int..

[72] Balthazart J., Foidart A., Surlemont C., Harada N., Naftolin F. (1992). Neuroanatomical specificity in the autoregulation of aromatase-immunoreactive neurons by androgens and estrogens: An immunocytochemical study. Brain Res..

[73] Bloch G., Gorski R. (1988). Cytoarchitectonic analysis of the SDN-POA of the intact and gonadectomized rat. J. Comp. Neurol..

[74] Simerly R., Swanson L., Chang C., Muramatsu M. (1990). Distribution of androgen and estrogen receptor mRNA-containing cells in the rat brain: An in situ hybridization study. J. Comp. Neurol..

[75] Rhees R.W., Shryne J.E., Gorski R.A. (1990). Termination of the hormone-sensitive period for differentiation of the sexually dimorphic nucleus of the preoptic area in male and female rats. Dev. Brain Res..

[76] Sanghera M.K., Simpson E.R., McPHAUL M.J., Kozlowski G., Conley A.J., Lephart E.D. (1991). Immunocytochemical distribution of aromatase cytochrome P450 in the rat brain using peptide-generated polyclonal antibodies. Endocrinology.

[77] Balthazart J., Foidart A., Surlemont C., Harada N. (1991). Distribution of aromatase-immunoreactive cells in the mouse forebrain. Cell Tissue Res..

[78] Balthazart J., Foidart A., Surlemont C., Harada N. (1991). Neuroanatomical specificity in the co-localization of aromatase and estrogen receptors. J. Neurobiol..

[79] Dellovade T., Rissman E., Thompson N., Harada N., Ottinger M. (1995). Co-localization of aromatase enzyme and estrogen receptor immunoreactivity in the preoptic area during reproductive aging. Brain Res..

[80] Nagano M., Shinoda K. (1994). Coexistence of the stigmoid body and estrogen receptor in some neuronal groups involved in rat reproductive functions. Brain Res..

[81] Rizvi T.A., Ennis M., Shipley M.T. (1992). Reciprocal connections between the medial preoptic area and the midbrain periaqueductal gray in rat: A WGA-HRP and PHA-L study. J. Comp. Neurol..

[82] Paut-Pagano L., Roky R., Valatx J.L., Kitahama K., Jouvet M. (1993). Anatomical distribution of prolactin-like immunoreactivity in the rat brain. Neuroendocrinology.

[83] Bridges R.S., Mann P.E. (1994). Prolactin-brain interactions in the induction of maternal behavior in rats. Psychoneuroendocrinology.

[84] Numan M. (1994). A neural circuitry analysis of maternal behavior in the rat. Acta Paediatr..

[85] Roky R., Paut-Pagano L., Goffin V., Kitahama K., Valatx J.L., Kelly P.A., Jouvet M. (1996). Distribution of prolactin receptors in the rat forebrain. Neuroendocrinology.

[86] Bakowska J.C., Morrell J.I. (1997). Atlas of the neurons that express mRNA for the long form of the prolactin receptor in the forebrain of the female rat. J. Comp. Neurol..

[87] Bole-Feysot C., Goffin V., Edery M., Binart N., Kelly P.A. (1998). Prolactin (PRL) and its receptor: Actions, signal transduction pathways and phenotypes observed in PRL receptor knockout mice. Endocr. Rev..

[88] Pi X.J., Grattan D.R. (1998). Distribution of prolactin receptor immunoreactivity in the brain of estrogen-treated, ovariectomized rats. J. Comp. Neurol..

[89] Pi X., Grattan D. (1999). Increased expression of both short and long forms of prolactin receptor mRNA in hypothalamic nuclei of lactating rats. J. Mol. Endocrinol..

[90] Argiolas A., Gessa G.L. (1991). Central functions of oxytocin. Neurosci. Biobehav. Rev..

[91] Crowley W.R., Armstrong W.E. (1992). Neurochemical regulation of oxytocin secretion in lactation. Endocr. Rev..

[92] Yoshimura R., Kiyama H., Kimura T., Araki T., Maeno H., Tanizawa O., Tohyama M. (1993). Localization of oxytocin receptor messenger ribonucleic acid in the rat brain. Endocrinology.

[93] Pedersen C.A., Caldwell J.D., Walker C., Ayers G., Mason G.A. (1994). Oxytocin activates the postpartum onset of rat maternal behavior in the ventral tegmental and medial preoptic areas. Behav. Neurosci..

[94] Corodimas K.P., Rosenblatt J.S., Morrell J.I. (1992). The habenular complex mediates hormonal stimulation of maternal behavior in rats. Behav. Neurosci..

[95] Corodimas K.P., Rosenblatt J.S., Canfield M.E., Morrell J.I. (1993). Neurons in the lateral subdivision of the habenular complex mediate the hormonal onset of maternal behavior in rats. Behav. Neurosci..

[96] Matthews-Felton T., Corodimas K.P., Rosenblatt J.S., Morrell J.I. (1995). Lateral habenula neurons are necessary for the hormonal onset of maternal behavior and for the display of postpartum estrus in naturally parturient female rats. Behav. Neurosci..

[97] Fleming A.S., Suh E.J., Korsmit M., Rusak B. (1994). Activation of Fos-like immunoreactivity in the medial preoptic area and limbic structures of maternal and social interactions in rats. Behav. Neurosci..

[98] Kirkpatrick B., Kim J.W., Insel T.R. (1994). Limbic system fos expression associated with paternal behavior. Brain Res..

[99] Lonstein J., Simmons D., Swann J., Stern J. (1997). Forebrain expression of c-fos due to active maternal behaviour in lactating rats. Neuroscience.

[100] Numan M., Numan M.J., Marzella S.R., Palumbo A. (1998). Expression of c-fos, fos B, and egr-1 in the medial preoptic area and bed nucleus of the stria terminalis during maternal behavior in rats. Brain Res..

[101] Li C., Chen P., Smith M. (1999). Neural populations in the rat forebrain and brainstem activated by the suckling stimulus as demonstrated by cFos expression. Neuroscience.

[102] Kalinichev M., Rosenblatt J.S., Nakabeppu Y., Morrell J.I. (2000). Induction of c-fos-like and fosB-like immunoreactivity reveals forebrain neuronal populations involved differentially in pup-mediated maternal behavior in juvenile and adult rats. J. Comp. Neurol..

[103] Morgan J.I., Curran T. (1991). Stimulus-transcription coupling in the nervous system: Involvement of the inducible proto-oncogenes fos and jun. Annu. Rev. Neurosci..

[104] Hoffman G.E., Lee W.S., Smith M.S., Abbud R. (1993). C-Fos and Fos-Related Antigens as Markers for Neuronal Activity: Perspectives From Neuroendocrine. Act. Immed. Early Genes Drugs Abus..

[105] Hoffman G.E., Smith M.S., Verbalis J.G. (1993). c-Fos and related immediate early gene products as markers of activity in neuroendocrine systems. Front. Neuroendocrinol..

[106] Bridges R.S. (1996). Biochemical basis of parental behavior in the rat. Advances in the Study of Behavior.

[107] Stack E.C., Numan M. (2000). The temporal course of expression of c-Fos and Fos B within the medial preoptic area and other brain regions of postpartum female rats during prolonged mother–young interactions. Behav. Neurosci..

[108] Lin S.H., Miyata S., Weng W., Matsunaga W., Ichikawa J., Furuya K., Nakashima T., Kiyohara T. (1998). Comparison of the expression of two immediate early gene proteins, FosB and Fos in the rat preoptic area, hypothalamus and brainstem during pregnancy, parturition and lactation. Neurosci. Res..

[109] Walsh C.J., Fleming A.S., Lee A., Magnusson J.E. (1996). The effects of olfactory and somatosensory desensitization on Fos-like immunoreactivity in the brains of pup-exposed postpartum rats. Behav. Neurosci..

[110] Lonstein J., De Vries G. (2000). Maternal behaviour in lactating rats stimulates c-fos in glutamate decarboxylase-synthesizing neurons of the medial preoptic area, ventral bed nucleus of the stria terminalis, and ventrocaudal periaqueductal gray. Neuroscience.

[111] Bridges R., Mann P., Coppeta J. (1999). Hypothalamic involvement in the regulation of maternal behaviour in the rat: Inhibitory roles for the ventromedial hypothalamus and the dorsal/anterior hypothalamic areas. J. Neuroendocrinol..

[112] Sheehan T.P., Cirrito J., Numan M.J., Numan M. (2000). Using c-Fos immunocytochemistry to identify forebrain regions that may inhibit maternal behavior in rats. Behav. Neurosci..

[113] Komisaruk B.R., Rosenblatt J.S., Barona M.L., Chinapen S., Nissanov J., T O’Bannon III R., Johnson B.M., Del Cerro M.C.R. (2000). Combined c-fos and 14C-2-deoxyglucose method to differentiate site-specific excitation from disinhibition: Analysis of maternal behavior in the rat. Brain Res..

[114] Lonstein J.S., Gréco B., De Vries G.J., Stern J.M., Blaustein J.D. (2000). Maternal behavior stimulates c-fos activity within estrogen receptor alpha-containing neurons in lactating rats. Neuroendocrinology.

[115] Sheehan T., Numan M. (2002). Estrogen, progesterone, and pregnancy termination alter neural activity in brain regions that control maternal behavior in rats. Neuroendocrinology.

[116] Insel T.R. (1990). Regional induction of c-fos-like protein in rat brain after estradiol administration. Endocrinology.

[117] Cattaneo E., Maggi A. (1990). C-fos induction by estrogen in specific rat brain areas. Eur. J. Pharmacol. Mol. Pharmacol..

[118] Mayer A.D., Monroy M.A., Rosenblatt J.S. (1990). Prolonged estrogen-progesterone treatment of nonpregnant ovariectomized rats: Factors stimulating home-cage and maternal aggression and short-latency maternal behavior. Horm. Behav..

[119] Grattan D.R. (2001). The actions of prolactin in the brain during pregnancy and lactation. Prog. Brain Res..

[120] Gammie S.C. (2005). Current models and future directions for understanding the neural circuitries of maternal behaviors in rodents. Behav. Cogn. Neurosci. Rev..

[121] Arrati P.G., Carmona C., Dominguez G., Beyer C., Rosenblatt J.S. (2006). GABA receptor agonists in the medial preoptic area and maternal behavior in lactating rats. Physiol. Behav..

[122] Olazabal D.E., Young L.J. (2006). Oxytocin receptors in the nucleus accumbens facilitate “spontaneous” maternal behavior in adult female prairie voles. Neuroscience.

[123] Broad K., Lévy F., Evans G., Kimura T., Keverne E., Kendrick K. (1999). Previous maternal experience potentiates the effect of parturition on oxytocin receptor mRNA expression in the paraventricular nucleus. Eur. J. Neurosci..

[124] Francis D.D., Champagne F.C., Meaney M.J. (2000). Variations in maternal behaviour are associated with differences in oxytocin receptor levels in the rat. J. Neuroendocrinol..

[125] Champagne F., Diorio J., Sharma S., Meaney M.J. (2001). Naturally occurring variations in maternal behavior in the rat are associated with differences in estrogen-inducible central oxytocin receptors. Proc. Natl. Acad. Sci. USA.

[126] Lightman S.L., Windle R.J., Wood S.A., Kershaw Y.M., Shanks N., Ingram C.D. (2001). Peripartum plasticity within the hypothalamo-pituitary-adrenal axis. Prog. Brain Res..

[127] Silva M.R.P., Bernardi M.M., Cruz-Casallas P.E., Felicio L.F. (2003). Pimozide injections into the nucleus accumbens disrupt maternal behaviour in lactating rats. Pharmacol. Toxicol..

[128] Numan M., Numan M.J., Pliakou N., Stolzenberg D.S., Mullins O.J., Murphy J.M., Smith C.D. (2005). The effects of D1 or D2 dopamine receptor antagonism in the medial preoptic area, ventral pallidum, or nucleus accumbens on the maternal retrieval response and other aspects of maternal behavior in rats. Behav. Neurosci..

[129] Miller S.M., Lonstein J.S. (2005). Dopamine d1 and d2 receptor antagonism in the preoptic area produces different effects on maternal behavior in lactating rats. Behav. Neurosci..

[130] Li M., Fleming A.S. (2003). The nucleus accumbens shell is critical for normal expression of pup-retrieval in postpartum female rats. Behav. Brain Res..

[131] Lee A., Li M., Watchus J., Fleming A.S. (1999). Neuroanatomical basis of maternal memory in postpartum rats: Selective role for the nucleus accumbens. Behav. Neurosci..

[132] Li M., Fleming A.S. (2003). Differential involvement of nucleus accumbens shell and core subregions in maternal memory in postpartum female rats. Behav. Neurosci..

[133] Lonstein J.S. (2002). Effects of dopamine receptor antagonism with haloperidol on nurturing behavior in the biparental prairie vole. Pharmacol. Biochem. Behav..

[134] Afonso V.M., King S., Chatterjee D., Fleming A.S. (2009). Hormones that increase maternal responsiveness affect accumbal dopaminergic responses to pup-and food-stimuli in the female rat. Horm. Behav..

[135] Vernotica E.M., Rosenblatt J.S., Morrell J.I. (1999). Microinfusion of cocaine into the medial preoptic area or nucleus accumbens transiently impairs maternal behavior in the rat. Behav. Neurosci..

[136] Insel T.R. (2003). Is social attachment an addictive disorder?. Physiol. Behav..

[137] Ferris C.F., Kulkarni P., Sullivan J.M., Harder J.A., Messenger T.L., Febo M. (2005). Pup suckling is more rewarding than cocaine: Evidence from functional magnetic resonance imaging and three-dimensional computational analysis. J. Neurosci..

[138] Seip K.M., Morrell J.I. (2007). Increasing the incentive salience of cocaine challenges preference for pup-over cocaine-associated stimuli during early postpartum: Place preference and locomotor analyses in the lactating female rat. Psychopharmacology.

[139] Stolzenberg D.S., McKenna J.B., Keough S., Hancock R., Numan M.J., Numan M. (2007). Dopamine D1 receptor stimulation of the nucleus accumbens or the medial preoptic area promotes the onset of maternal behavior in pregnancy-terminated rats. Behav. Neurosci..

[140] Numan M., Stolzenberg D.S. (2008). Hypothalamic interaction with the mesolimbic dopamine system and the regulation of maternal responsiveness. Neurobiology of the Parental Brain.

[141] Pereira M., Ferreira A. (2006). Demanding pups improve maternal behavioral impairments in sensitized and haloperidol-treated lactating female rats. Behav. Brain Res..

[142] Bardo M.T., Donohew R., Harrington N.G. (1996). Psychobiology of novelty seeking and drug seeking behavior. Behav. Brain Res..

[143] Berridge K.C., Robinson T.E. (1998). What is the role of dopamine in reward: Hedonic impact, reward learning, or incentive salience?. Brain Res. Rev..

[144] Ikemoto S., Panksepp J. (1999). The role of nucleus accumbens dopamine in motivated behavior: A unifying interpretation with special reference to reward-seeking. Brain Res. Rev..

[145] Becker J.B. (1999). Gender differences in dopaminergic function in striatum and nucleus accumbens. Pharmacol. Biochem. Behav..

[146] Hunt G.E., McGregor I.S. (2002). Contrasting effects of dopamine antagonists and frequency reduction on Fos expression induced by lateral hypothalamic stimulation. Behav. Brain Res..

[147] Reynolds S.M., Berridge K.C. (2002). Positive and negative motivation in nucleus accumbens shell: Bivalent rostrocaudal gradients for GABA-elicited eating, taste “liking”/“disliking” reactions, place preference/avoidance, and fear. J. Neurosci..

[148] Horvitz J.C. (2002). Dopamine gating of glutamatergic sensorimotor and incentive motivational input signals to the striatum. Behav. Brain Res..

[149] Olazabal D., Abercrombie E., Rosenblatt J., Morrell J. (2004). The content of dopamine, serotonin, and their metabolites in the neural circuit that mediates maternal behavior in juvenile and adult rats. Brain Res. Bull..

[150] Bosch O., Pförtsch J., Beiderbeck D., Landgraf R., Neumann I. (2010). Maternal behaviour is associated with vasopressin release in the medial preoptic area and bed nucleus of the stria terminalis in the rat. J. Neuroendocrinol..

[151] Anderson G.M., Grattan D.R., van den Ancker W., Bridges R.S. (2006). Reproductive experience increases prolactin responsiveness in the medial preoptic area and arcuate nucleus of female rats. Endocrinology.

[152] Bridges R.S., Robertson M.C., Shiu R.P., Sturgis J.D., Henriquez B.M., Mann P.E. (1997). Central lactogenic regulation of maternal behavior in rats: Steroid dependence, hormone specificity, and behavioral potencies of rat prolactin and rat placental lactogen I. Endocrinology.

[153] Russell J.A., Douglas A.J., Ingram C.D. (2001). Brain preparations for maternity—adaptive changes in behavioral and neuroendocrine systems during pregnancy and lactation. An overview. Prog. Brain Res..

[154] Bridges R.S., Rigero B.A., Byrnes E.M., Yang L., Walker A.M. (2001). Central infusions of the recombinant human prolactin receptor antagonist, S179D-PRL, delay the onset of maternal behavior in steroid-primed, nulliparous female rats. Endocrinology.

[155] Leckman J.F., Herman A.E. (2002). Maternal behavior and developmental psychopathology. Biol. Psychiatry.

[156] Torner L., Toschi N., Nava G., Clapp C., Neumann I.D. (2002). Increased hypothalamic expression of prolactin in lactation: Involvement in behavioural and neuroendocrine stress responses. Eur. J. Neurosci..

[157] Wettschureck N., Moers A., Hamalainen T., Lemberger T., Schütz G., Offermanns S. (2004). Heterotrimeric G proteins of the Gq/11 family are crucial for the induction of maternal behavior in mice. Mol. Cell. Biol..

[158] Bridges R.S., Hays L.E. (2005). Steroid-induced alterations in mRNA expression of the long form of the prolactin receptor in the medial preoptic area of female rats: Effects of exposure to a pregnancy-like regimen of progesterone and estradiol. Mol. Brain Res..

[159] Grattan D.R., Steyn F.J., Kokay I.C., Anderson G.M., Bunn S.J. (2008). Pregnancy-induced adaptation in the neuroendocrine control of prolactin secretion. J. Neuroendocrinol..

[160] Gammie S.C., Nelson R.J. (1999). Maternal aggression is reduced in neuronal nitric oxide synthase-deficient mice. J. Neurosci..

[161] Lonstein J.S., Gammie S.C. (2002). Sensory, hormonal, and neural control of maternal aggression in laboratory rodents. Neurosci. Biobehav. Rev..

[162] Gammie S.C., Negron A., Newman S.M., Rhodes J.S. (2004). Corticotropin-releasing factor inhibits maternal aggression in mice. Behav. Neurosci..

[163] Popeski N., Woodside B. (2004). Central nitric oxide synthase inhibition disrupts maternal behavior in the rat. Behav. Neurosci..

[164] Numan M. (2004). Maternal behaviors: Central integration or independent parallel circuits? Theoretical comment on Popeski and Woodside (2004). Behav. Neurosci..

[165] Bosch O.J., Meddle S.L., Beiderbeck D.I., Douglas A.J., Neumann I.D. (2005). Brain oxytocin correlates with maternal aggression: Link to anxiety. J. Neurosci..

[166] Lonstein J., Dominguez J., Putnam S., De Vries G., Hull E. (2003). Intracellular preoptic and striatal monoamines in pregnant and lactating rats: Possible role in maternal behavior. Brain Res..

[167] Miller S.M., Lonstein J.S. (2009). Dopaminergic projections to the medial preoptic area of postpartum rats. Neuroscience.

[168] Numan M., Numan M.J. (1997). Projection sites of medial preoptic area and ventral bed nucleus of the stria terminalis neurons that express Fos during maternal behavior in female rats. J. Neuroendocrinol..

[169] Kudo T., Uchigashima M., Miyazaki T., Konno K., Yamasaki M., Yanagawa Y., Minami M., Watanabe M. (2012). Three types of neurochemical projection from the bed nucleus of the stria terminalis to the ventral tegmental area in adult mice. J. Neurosci..

[170] Jennings J.H., Sparta D.R., Stamatakis A.M., Ung R.L., Pleil K.E., Kash T.L., Stuber G.D. (2013). Distinct extended amygdala circuits for divergent motivational states. Nature.

[171] Numan M., Stolzenberg D.S., Dellevigne A.A., Correnti C.M., Numan M.J. (2009). Temporary inactivation of ventral tegmental area neurons with either muscimol or baclofen reversibly disrupts maternal behavior in rats through different underlying mechanisms. Behav. Neurosci..

[172] Seip K.M., Morrell J.I. (2009). Transient inactivation of the ventral tegmental area selectively disrupts the expression of conditioned place preference for pup-but not cocaine-paired contexts. Behav. Neurosci..

[173] Febo M., Felix-Ortiz A.C., Johnson T.R. (2010). Inactivation or inhibition of neuronal activity in the medial prefrontal cortex largely reduces pup retrieval and grouping in maternal rats. Brain Res..

[174] Numan M., Woodside B. (2010). Maternity: Neural mechanisms, motivational processes, and physiological adaptations. Behav. Neurosci..

[175] Mattson B.J., Williams S.E., Rosenblatt J.S., Morrell J.I. (2003). Preferences for cocaine-or pup-associated chambers differentiates otherwise behaviorally identical postpartum maternal rats. Psychopharmacology.

[176] Curtis J.T., Liu Y., Aragona B.J., Wang Z. (2006). Dopamine and monogamy. Brain Res..

[177] Perrin G., Meurisse M., Lévy F. (2007). Inactivation of the medial preoptic area or the bed nucleus of the stria terminalis differentially disrupts maternal behavior in sheep. Horm. Behav..

[178] Rutherford H., Williams S., Moy S., Mayes L., Johns J. (2011). Disruption of maternal parenting circuitry by addictive process: Rewiring of reward and stress systems. Front. Psychiatry.

[179] Lavi-Avnon Y., Weller A., Finberg J.P., Gispan-Herman I., Kinor N., Stern Y., Schroeder M., Gelber V., Bergman S.Y., Overstreet D.H. (2008). The reward system and maternal behavior in an animal model of depression: A microdialysis study. Psychopharmacology.

[180] Stolzenberg D.S., Numan M. (2011). Hypothalamic interaction with the mesolimbic DA system in the control of the maternal and sexual behaviors in rats. Neurosci. Biobehav. Rev..

[181] Mattson B., Morrell J. (2005). Preference for cocaine-versus pup-associated cues differentially activates neurons expressing either Fos or cocaine-and amphetamine-regulated transcript in lactating, maternal rodents. Neuroscience.

[182] Wansaw M.P., Pereira M., Morrell J.I. (2008). Characterization of maternal motivation in the lactating rat: Contrasts between early and late postpartum responses. Horm. Behav..

[183] Pereira M., Morrell J.I. (2010). The medial preoptic area is necessary for motivated choice of pup-over cocaine-associated environments by early postpartum rats. Neuroscience.

[184] Dölen G., Darvishzadeh A., Huang K.W., Malenka R.C. (2013). Social reward requires coordinated activity of nucleus accumbens oxytocin and serotonin. Nature.

[185] Rilling J.K. (2013). The neural and hormonal bases of human parentalcare. Neuropsychologia.

[186] Stolzenberg D.S., Rissman E.F. (2011). Oestrogen-independent, experience-induced maternal behaviour in female mice. J. Neuroendocrinol..

[187] Afonso V.M., Grella S.L., Chatterjee D., Fleming A.S. (2008). Previous maternal experience affects accumbal dopaminergic responses to pup-stimuli. Brain Res..

[188] Parada M., King S., Li M., Fleming A.S. (2008). The roles of accumbal dopamine D1 and D2 receptors in maternal memory in rats. Behav. Neurosci..

[189] Olazábal D.E., Pereira M., Agrati D., Ferreira A., Fleming A.S., González-Mariscal G., Lévy F., Lucion A.B., Morrell J.I., Numan M. (2013). Flexibility and adaptation of the neural substrate that supports maternal behavior in mammals. Neurosci. Biobehav. Rev..

[190] Pereira M., Morrell J.I. (2009). The changing role of the medial preoptic area in the regulation of maternal behavior across the postpartum period: Facilitation followed by inhibition. Behav. Brain Res..

[191] Rilling J.K., Young L.J. (2014). The biology of mammalian parenting and its effect on offspring social development. Science.

[192] Lonstein J.S., Lévy F., Fleming A.S. (2015). Common and divergent psychobiological mechanisms underlying maternal behaviors in non-human and human mammals. Horm. Behav..

[193] McHenry J.A., Rubinow D.R., Stuber G.D. (2015). Maternally responsive neurons in the bed nucleus of the stria terminalis and medial preoptic area: Putative circuits for regulating anxiety and reward. Front. Neuroendocrinol..

[194] Brunton P.J., Russell J.A. (2008). The expectant brain: Adapting for motherhood. Nat. Rev. Neurosci..

[195] Schiller C.E., O’Hara M.W., Rubinow D.R., Johnson A.K. (2013). Estradiol modulates anhedonia and behavioral despair in rats and negative affect in a subgroup of women at high risk for postpartum depression. Physiol. Behav..

[196] Walker D.L., Toufexis D.J., Davis M. (2003). Role of the bed nucleus of the stria terminalis versus the amygdala in fear, stress, and anxiety. Eur. J. Pharmacol..

[197] Nephew B.C., Bridges R.S. (2008). Arginine vasopressin V1a receptor antagonist impairs maternal memory in rats. Physiol. Behav..

[198] d’Cunha T., King S., Fleming A., Lévy F. (2011). Oxytocin receptors in the nucleus accumbens shell are involved in the consolidation of maternal memory in postpartum rats. Horm. Behav..

[199] Scanlan V.F., Byrnes E.M., Bridges R.S. (2006). Reproductive experience and activation of maternal memory. Behav. Neurosci..

[200] Vecsey C.G., Hawk J.D., Lattal K.M., Stein J.M., Fabian S.A., Attner M.A., Cabrera S.M., McDonough C.B., Brindle P.K., Abel T. (2007). Histone deacetylase inhibitors enhance memory and synaptic plasticity via CREB: CBP-dependent transcriptional activation. J. Neurosci..

[201] Brusco J., Wittmann R., de Azevedo M.S., Lucion A.B., Franci C.R., Giovenardi M., Rasia-Filho A.A. (2008). Plasma hormonal profiles and dendritic spine density and morphology in the hippocampal CA1 stratum radiatum, evidenced by light microscopy, of virgin and postpartum female rats. Neurosci. Lett..

[202] Kim P., Leckman J.F., Mayes L.C., Feldman R., Wang X., Swain J.E. (2010). The plasticity of human maternal brain: Longitudinal changes in brain anatomy during the early postpartum period. Behav. Neurosci..

[203] Leuner B., Gould E. (2010). Dendritic growth in medial prefrontal cortex and cognitive flexibility are enhanced during the postpartum period. J. Neurosci..

[204] Shams S., Pawluski J.L., Chatterjee-Chakraborty M., Oatley H., Mastroianni A., Fleming A.S. (2012). Dendritic morphology in the striatum and hypothalamus differentially exhibits experience-dependent changes in response to maternal care and early social isolation. Behav. Brain Res..

[205] Pawluski J.L., Galea L.A. (2006). Hippocampal morphology is differentially affected by reproductive experience in the mother. J. Neurobiol..

[206] Pawluski J.L., Brummelte S., Barha C.K., Crozier T.M., Galea L.A. (2009). Effects of steroid hormones on neurogenesis in the hippocampus of the adult female rodent during the estrous cycle, pregnancy, lactation and aging. Front. Neuroendocrinol..

[207] Barrett J., Fleming A.S. (2011). Annual research review: All mothers are not created equal: Neural and psychobiological perspectives on mothering and the importance of individual differences. J. Child Psychol. Psychiatry.

[208] Moses-Kolko E.L., Fraser D., Wisner K.L., James J.A., Saul A.T., Fiez J.A., Phillips M.L. (2011). Rapid habituation of ventral striatal response to reward receipt in postpartum depression. Biol. Psychiatry.

[209] Agrati D., Zuluaga M., Fernández-Guasti A., Meikle A., Ferreira A. (2008). Maternal condition reduces fear behaviors but not the endocrine response to an emotional threat in virgin female rats. Horm. Behav..

[210] Slattery D.A., Neumann I.D. (2008). No stress please! Mechanisms of stress hyporesponsiveness of the maternal brain. J. Physiol..

[211] Bosch O.J. (2011). Maternal nurturing is dependent on her innate anxiety: The behavioral roles of brain oxytocin and vasopressin. Horm. Behav..

[212] Bosch O., Krömer S., Brunton P., Neumann I. (2004). Release of oxytocin in the hypothalamic paraventricular nucleus, but not central amygdala or lateral septum in lactating residents and virgin intruders during maternal defence. Neuroscience.

[213] Nephew B.C., Bridges R.S. (2008). Central actions of arginine vasopressin and a V1a receptor antagonist on maternal aggression, maternal behavior, and grooming in lactating rats. Pharmacol. Biochem. Behav..

[214] Numan M. (2012). Maternal behavior: Neural circuits, stimulus valence, and motivational processes. Parenting.

[215] Takayanagi Y., Yoshida M., Bielsky I.F., Ross H.E., Kawamata M., Onaka T., Yanagisawa T., Kimura T., Matzuk M.M., Young L.J. (2005). Pervasive social deficits, but normal parturition, in oxytocin receptor-deficient mice. Proc. Natl. Acad. Sci. USA.

[216] Consiglio A.R., Borsoi A., Pereira G.A., Lucion A.B. (2005). Effects of oxytocin microinjected into the central amygdaloid nucleus and bed nucleus of stria terminalis on maternal aggressive behavior in rats. Physiol. Behav..

[217] Pedersen C., Vadlamudi S., Boccia M., Amico J. (2006). Maternal behavior deficits in nulliparous oxytocin knockout mice. Genes Brain Behav..

[218] Champagne F.A., Meaney M.J. (2006). Stress during gestation alters postpartum maternal care and the development of the offspring in a rodent model. Biol. Psychiatry.

[219] Lonstein J.S. (2007). Regulation of anxiety during the postpartum period. Front. Neuroendocrinol..

[220] Bosch O.J., Neumann I.D. (2008). Brain vasopressin is an important regulator of maternal behavior independent of dams’ trait anxiety. Proc. Natl. Acad. Sci. USA.

[221] Leng G., Meddle S.L., Douglas A.J. (2008). Oxytocin and the maternal brain. Curr. Opin. Pharmacol..

[222] Veenema A.H., Neumann I.D. (2008). Central vasopressin and oxytocin release: Regulation of complex social behaviours. Prog. Brain Res..

[223] Neumann I.D. (2008). Brain oxytocin: A key regulator of emotional and social behaviours in both females and males. J. Neuroendocrinol..

[224] Bosch O.J., Neumann I.D. (2010). Vasopressin released within the central amygdala promotes maternal aggression. Eur. J. Neurosci..

[225] Neumann I.D., Landgraf R. (2012). Balance of brain oxytocin and vasopressin: Implications for anxiety, depression, and social behaviors. Trends Neurosci..

[226] Yoshihara C., Numan M., Kuroda K.O. (2017). Oxytocin and parental behaviors. Behavioral Pharmacology of Neuropeptides: Oxytocin.

[227] Landgraf R., Neumann I.D. (2004). Vasopressin and oxytocin release within the brain: A dynamic concept of multiple and variable modes of neuropeptide communication. Front. Neuroendocrinol..

[228] Beiderbeck D.I., Neumann I.D., Veenema A.H. (2007). Differences in intermale aggression are accompanied by opposite vasopressin release patterns within the septum in rats bred for low and high anxiety. Eur. J. Neurosci..

[229] Caldwell H.K., Lee H.J., Macbeth A.H., Young III W.S. (2008). Vasopressin: Behavioral roles of an “original” neuropeptide. Prog. Neurobiol..

[230] McGregor I., Callaghan P., Hunt G. (2008). From ultrasocial to antisocial: A role for oxytocin in the acute reinforcing effects and long-term adverse consequences of drug use?. Br. J. Pharmacol..

[231] Carter C.S. (2014). Oxytocin pathways and the evolution of human behavior. Annu. Rev. Psychol..

[232] Knobloch H.S., Grinevich V. (2014). Evolution of oxytocin pathways in the brain of vertebrates. Front. Behav. Neurosci..

[233] Febo M., Numan M., Ferris C.F. (2005). Functional magnetic resonance imaging shows oxytocin activates brain regions associated with mother–pup bonding during suckling. J. Neurosci..

[234] Febo M., Stolberg T.L., Numan M., Bridges R.S., Kulkarni P., Ferris C.F. (2008). Nursing stimulation is more than tactile sensation: It is a multisensory experience. Horm. Behav..

[235] Febo M. (2011). A bold view of the lactating brain: Functional magnetic resonance imaging studies of suckling in awake dams. J. Neuroendocrinol..

[236] Dobolyi A., Grattan D., Stolzenberg D. (2014). Preoptic inputs and mechanisms that regulate maternal responsiveness. J. Neuroendocrinol..

[237] Gammie S.C., Hasen N.S., Awad T.A., Auger A.P., Jessen H.M., Panksepp J.B., Bronikowski A.M. (2005). Gene array profiling of large hypothalamic CNS regions in lactating and randomly cycling virgin mice. Mol. Brain Res..

[238] Stolzenberg D.S., Stevens J.S., Rissman E.F. (2012). Experience-facilitated improvements in pup retrieval; evidence for an epigenetic effect. Horm. Behav..

[239] Akbari E.M., Shams S., Belay H.T., Kaiguo M., Razak Z., Kent C.F., Westwood T., Sokolowski M.B., Fleming A.S. (2013). The effects of parity and maternal behavior on gene expression in the medial preoptic area and the medial amygdala in postpartum and virgin female rats: A microarray study. Behav. Neurosci..

[240] Roozendaal B., Hernandez A., Cabrera S.M., Hagewoud R., Malvaez M., Stefanko D.P., Haettig J., Wood M.A. (2010). Membrane-associated glucocorticoid activity is necessary for modulation of long-term memory via chromatin modification. J. Neurosci..

[241] Malvaez M., Sanchis-Segura C., Vo D., Lattal K.M., Wood M.A. (2010). Modulation of chromatin modification facilitates extinction of cocaine-induced conditioned place preference. Biol. Psychiatry.

[242] Wynne-Edwards K.E., Timonin M.E. (2007). Paternal care in rodents: Weakening support for hormonal regulation of the transition to behavioral fatherhood in rodent animal models of biparental care. Horm. Behav..

[243] Kenkel W., Paredes J., Yee J., Pournajafi-Nazarloo H., Bales K., Carter C. (2012). Neuroendocrine and behavioural responses to exposure to an infant in male prairie voles. J. Neuroendocrinol..

[244] De Jong T.R., Chauke M., Harris B.N., Saltzman W. (2009). From here to paternity: Neural correlates of the onset of paternal behavior in California mice (*Peromyscus californicus*). Horm. Behav..

[245] De Jong T., Measor K., Chauke M., Harris B., Saltzman W. (2010). Brief pup exposure induces Fos expression in the lateral habenula and serotonergic caudal dorsal raphe nucleus of paternally experienced male California mice (*Peromyscus californicus*). Neuroscience.

[246] Lambert K.G., Franssen C.L., Bardi M., Hampton J.E., Hainley L., Karsner S., Tu E.B., Hyer M.M., Crockett A., Baranova A. (2011). Characteristic neurobiological patterns differentiate paternal responsiveness in two *Peromyscus* species. Brain Behav. Evol..

[247] Tachikawa K.S., Yoshihara Y., Kuroda K.O. (2013). Behavioral transition from attack to parenting in male mice: A crucial role of the vomeronasal system. J. Neurosci..

[248] Isogai Y., Wu Z., Love M.I., Ahn M.H.Y., Bambah-Mukku D., Hua V., Farrell K., Dulac C. (2018). Multisensory logic of infant-directed aggression by males. Cell.

[249] Tsuneoka Y., Yoshida S., Takase K., Oda S., Kuroda M., Funato H. (2017). Neurotransmitters and neuropeptides in gonadal steroid receptor-expressing cells in medial preoptic area subregions of the male mouse. Sci. Rep..

[250] Numan M., Young L.J. (2016). Neural mechanisms of mother–infant bonding and pair bonding: Similarities, differences, and broader implications. Horm. Behav..

[251] Numan M. (2017). Parental Behavior. Reference Module in Neuroscience and Biobehavioral Psychology.

[252] Chen C. (2006). CiteSpace II: Detecting and visualizing emerging trends and transient patterns in scientific literature. J. Am. Soc. Inf. Sci. Technol..

[253] Haddaway N.R., Collins A.M., Coughlin D., Kirk S. (2015). The role of Google Scholar in evidence reviews and its applicability to grey literature searching. PLoS ONE.

[254] Kim S., Soeken T.A., Cromer S.J., Martinez S.R., Hardy L.R., Strathearn L. (2014). Oxytocin and postpartum depression: Delivering on what’s known and what’s not. Brain Res..

